# Self-Conscious Emotions and the Right Fronto-Temporal and Right Temporal Parietal Junction

**DOI:** 10.3390/brainsci12020138

**Published:** 2022-01-20

**Authors:** Adriana LaVarco, Nathira Ahmad, Qiana Archer, Matthew Pardillo, Ray Nunez Castaneda, Anthony Minervini, Julian Paul Keenan

**Affiliations:** Cognitive Neuroimaging Lab, Montclair State University, Montclair, 07043 NJ, USA; lavarcoa1@mail.montclair.edu (A.L.); ahmadn3@mail.montclair.edu (N.A.); archerq1@mail.montclair.edu (Q.A.); pardillom1@montclair.edu (M.P.); nunezcastanr1@montclair.edu (R.N.C.); minervinia2@montclair.edu (A.M.)

**Keywords:** self-conscious emotions, self-awareness, rTPJ, fronto-temporal, emotions, laterality

## Abstract

For more than two decades, research focusing on both clinical and non-clinical populations has suggested a key role for specific regions in the regulation of self-conscious emotions. It is speculated that both the expression and the interpretation of self-conscious emotions are critical in humans for action planning and response, communication, learning, parenting, and most social encounters. Empathy, Guilt, Jealousy, Shame, and Pride are all categorized as self-conscious emotions, all of which are crucial components to one’s sense of self. There has been an abundance of evidence pointing to the right Fronto-Temporal involvement in the integration of cognitive processes underlying the expression of these emotions. Numerous regions within the right hemisphere have been identified including the right temporal parietal junction (rTPJ), the orbitofrontal cortex (OFC), and the inferior parietal lobule (IPL). In this review, we aim to investigate patient cases, in addition to clinical and non-clinical studies. We also aim to highlight these specific brain regions pivotal to the right hemispheric dominance observed in the neural correlates of such self-conscious emotions and provide the potential role that self-conscious emotions play in evolution.

## 1. Introduction

Empathy, Guilt, Jealousy, Shame, and Pride are categorized as self-conscious emotions as they require a degree of self-awareness to be experienced [1]. For example, one might feel *prideful* after winning a competition, *guilty* after stealing a candy bar from a gas station, or *embarrassed* after answering a question wrong in front of the class. It is thought that self-conscious emotions are reliant on an individual being self-aware and having thoughts related to their own specific behavior and/or cognition [1]. One’s self is generally being contrasted in these emotional states (i.e., the experiencing of self-conscious emotions) with either a different self version or another person’s self. 

It is speculated that these emotions are critical for the social complexity that has come to symbolize human societies. While all emotions provide survival benefits, self-conscious emotions are thought to modify one’s own thinking and behavior to adjust, replicate, accentuate, or extinguish such cognitions and actions as the environment changes. That is, one’s sense of self is modified as the demands of any given situation changes. Experiencing guilt should reduce, for example, anti-social behavior while empathy should allow one to interact more successfully with others. These abilities have built a unique social system for humankind, differing significantly from other tightly bonded animal systems such as those witnessed in birds, fish, eusocial insects, etc.

One’s sense of self, self-awareness, and self-image are mediated primarily by structures that are right lateralized, though there is subtle involvement of medial regions as well [2,3,4]. An individual’s mental state attribution, which is the ability to recognize one’s own and other individuals’ mental states, is also dominated by right hemisphere activity, mainly involving the rTPJ [5]. Damage to the right hemisphere, Neurostimulation, and disorders that disrupt the function of the rTPJ and right Fronto-Temporal structures can cause disruptions of one’s sense of self and/or result in a complete lack of self [2]. 

A number of researchers are credited for highlighting the importance of self-conscious emotions as well as providing a timeline for their development [6,7]. At approximately one year old, children begin formulating their sense of self [7]. Once children develop their sense of self, they can then begin setting standards and rules, which in turn develops their expression of self-conscious emotions [7]. This can occur anywhere between the ages of two and three years old. While self-evaluation begins early on in life, the exposure to positive and negative social interactions and behaviors have an effect on an individual’s expression of self-conscious emotions [8]. The work noted here stems from Michael Lewis, and similar to Gallup’s mirror-test, Lewis has demonstrated that there is a correlation between self-conscious emotions and self-awareness. His work provides a measure of self-awareness that has proven extremely valuable. 

It is evident that these emotions have a distinct tie to self-awareness, though that issue will not be examined here (see above in terms of Lewis). With lateralized differences being well known in the human brain [9], our goal here is to highlight that the right Fronto-Temporo and rTPJ are regions that specifically allow for the experience of and understanding of self-conscious emotions.

The experiencing of a self-conscious emotion almost always involves the experiencing of non-self-emotions. That is, jealousy is often anger, fear, rage etc., coupled with the notion that one’s self is ‘less than’ or the self has just ‘lost’. Shame is paired with sadness, while empathy may be associated with affiliative bonding. Typically, one’s reference to self-conscious emotions is the part of the emotion that involves the self-experiencing itself. That is, while fear may be an aspect of shame, by definition shame as a self-conscious emotion would be that aspect of the experience in which a person is ‘regretting’ or ‘thinking’ or ‘re-experiencing’ the moment of ‘I am sorry I did that’. This unique aspect of emotional processing is what is of interest here.

## 2. Empathy

The neural correlates and networks involved in the processes linked to social cognition, specifically self-conscious emotions, have been of interest for decades [10]. Evidence points to the involvement of the right hemisphere in social cognition and emotional processing; more specifically empathy and empathic responses, which are crucial for individual success in social interaction. This is likely due to the right hemisphere’s socio-cognitive abilities including facial recognition, self-awareness, and its role in the interpretation of emotional expression [11,12,13]. These are all key higher-cognitive processes responsible for social cognition, and self-conscious emotions are critical to successful social relationships and the navigation of social stimuli [14,15,16,17,18]. 

Self-awareness and self-other distinction, thought to be mediated by the right hemisphere [19], are important regarding emotional processing and empathy as they contribute to social cognitive processes [11]. Self-awareness allows individuals to distinguish between self and other; this understanding forms the foundation of empathy [12]. Empathy is obviously a multi-faceted, complex emotion that is critical to social interaction and success in social encounters. It enables one to perceive and understand the feelings and experiences of those around us, comprehending that information cognitively, and then allowing for appropriate responding, which forms the basis for much of our society [20].

Furthermore, research has suggested key regions located in the right hemisphere that are linked to empathy, including the right Temporal Parietal Junction (rTPJ) [13,21,22,23,24,25,26], the right ventromedial prefrontal cortex [27], the right ventral premotor area, and the right superior temporal gyrus [28], as well as the right somatosensory related cortices [17]. Case studies and brain stimulation have provided confirmation of correlational neuroimaging techniques [27,29,30]. In other words, the relationship is one of causality and without these structures, there is either a reduction, or absence, of empathy.

Empathizing with others is crucial to humans; it is a fundamental aspect of social cognition and behavior [24], as it is how we *affectively* recognize the feelings of others, connect, and build relationships. In addition to empathy, many would argue that Theory of Mind (ToM), which refers to the ability to attribute mental states (and is typically void of emotion), is just as important [31]. Numerous studies have demonstrated that there are some brain regions in the right hemisphere, including the rTPJ, which are responsible for carrying out functions regarding both empathy and ToM. ToM should not be mistaken for an emotion, and while there is an overlap in both empathy and ToM, research has found that they follow distinct neural networks. Empathy builds on ToM, as the ability to perceive the mental states of other individuals and recognize those social cues is very important. However, even though empathy relies on the inferring of the mental states of others, the emotional processing and responding to stimuli that follows is far more complicated [32,33].

It is well known that the rTPJ is linked to social cognition [34,35]. For example, Miller et al. [13] investigated the rTPJ’s exact role in the neurological basis of empathy to further understand how we process the suffering of others and how we handle such social situations. Participants first played a computer game that did not present any emotional stimuli and were then introduced to an emotionally neutral clip. They were additionally presented with a clip of the suffering and sadness of other individuals, such as a child experiencing severe grief. This was all carried out while disrupting the activity of the participants’ rTPJ utilizing TMS (Transcranial Magnetic Stimulation). Interestingly, activity of the parasympathetic nervous system was decreased following TMS to the rTPJ, which led to the conclusion that empathy follows a pathway that is being regulated by the rTPJ. The study demonstrated that the rTPJ also plays a role in autonomic and affective responses to others’ suffering. Young et al. [36] and Chou and Chen [25] additionally concluded that the rTPJ is involved in moral judgement, supporting a causal link between the rTPJ and its role in empathy. 

In addition to the rTPJ, right somatosensory cortices are also connected to empathetic cognitive processes, as they are involved in the judging of emotional states of others. These areas include the primary and secondary somatosensory cortices, the insula, and the anterior supramarginal gyrus. Adolphs et al. [17] analyzed these areas as well as the correlates and function of the right hemisphere in recognizing emotion through a quantitative study of 108 participants who suffered from focal brain injuries. The integration of the right somatosensory cortices was detected following three distinct tasks observing the participant’s ability to recognize and name six basic emotions. Interestingly, the right ventral premotor area and the right superior temporal gyrus have been researched regarding face and hand imitation. 

Mirror neurons may additionally serve a purpose in ‘feeling’ others’ emotions [28]. Shamay-Tsoory et al. [27] studied the empathic responses of 25 patients who had suffered brain damage to the prefrontal cortex, comparing these responses to patients with posterior lesions as well as healthy participants. Numerous patients that presented the greatest empathic response deficit were those who had lesions to their right ventromedial prefrontal cortex. These findings suggest that this area (identified for mirror-neuron involvement) is another crucial brain region responsible for integrating social stimuli as well as affecting and generating the appropriate empathic responses in specific situations.

There are cases where a deficit or even a lack of empathy is present due to brain injuries or the inactivity of specific regions due to certain disorders. These individuals are faced with great challenges in social interactions due to impairment of social cognition [32]. They are unable to understand and express feelings, either their own or those of others. Borderline and narcissistic personality disorders are very distinct examples of emotional dysregulation as individuals with these disorders lack the ability to empathize with others [37]. Pathological narcissism presents itself due to structural differences in brain regions of the right hemisphere. It is specifically attributed to decreased cortical thickness in the right dorsolateral prefrontal cortex (DLPFC) and right inferior frontal cortex, as well as decreased cortical volume in the right DLPFC and the right postcentral gyrus [38]. Once again, damage to the right ventromedial prefrontal cortex causes the absence of empathy in individuals, as seen in patients who suffer from brain injuries. This impaired empathetic response is only observed in individuals with lesions to the right hemisphere; left hemispheric damage has much less of an effect on empathy [27]. Lesions to the right hemisphere influence emotion recognition, which leads to the inability to integrate social and facial cues that allow empathetic responses [39].

## 3. Guilt

Guilt is a self-conscious emotion that employs numerous cognitive and emotional processes, but usually there are aspects involving violation of one’s moral beliefs or ‘code’. Abnormal guilty feelings have been observed in psychiatric conditions such as depression, obsessive-compulsive disorder (OCD), and antisocial disorder [40]. Guilt can also be found in individuals with post-traumatic stress disorder (PTSD) following exposure to a traumatic event [41]. Deception can increase guilt, and as many of us can attest, confessions primarily exist to relieve one’s inner cognitive dissonance [42]. 

The Guilty Knowledge Test (GKT) is a psychophysiological technique that measures the guilty knowledge an individual is harboring [43]. Jung et al. examined 30 healthy male participants in which each participant that entered the room was assigned an envelope, either innocent or guilty. The guilty group had to partake in a mock theft activity, while the innocent group performed a similar activity without the crime. EEG was employed to record the brain areas activated in each group. In the innocent group, there was activation in the temporal region and anterior cingulate. In the guilty group, the results revealed activation in the right middle frontal gyrus and right cingulate gyrus [44]. These results are similar to Karim et al., where 20 volunteers participated in theft role play. Every participant received one of two papers, “thief” or “innocent.” The assigned “thief” group had to partake in a mock theft activity while the innocent group waited. First, the thief group received cathodal transcranial direct-current stimulation (tDCS) aimed to suppress brain activity. Then, the participants underwent anodal tDCS, which is aimed to enhance cortical regional activity. Lastly, the group serving as controls were treated with sham stimulation. Participants were then asked to rate their feelings of guilt after the interrogation. Remarkably, inhibition of the right anterior prefrontal cortex by cathodal tDCS resulted in lower feelings of guilt while deceiving the interrogator [45]. The study suggested that cathodal tDCS can modulate guilt and supports the finding that the anterior prefrontal cortex (aPFC) and temporal regions play a role in deceptive behavior [44,46].

Guilt appears to be a factor that may increase the severity of OCD and negatively impact the treatment outcome of this disorder [47]. There are two types of guilt associated with OCD that have been identified: deontological guilt (DG), a transgression of inner values; and altruistic guilt (AG), a transgression of interpersonal situations [48]. Basile et al. found that patients have reduced activation in the anterior cingulate (ACC) and frontal gyrus when experiencing guilt, regardless of DG or AG. When separately considering each type of guilt (against each of their controls), patients showed decreased activation in the ACC, the right insula, and the precuneus for DG [49].

Hennig-Fast et al. examined twenty participants with OCD and twenty healthy controls. To assess the interpersonal feelings of guilt, a questionnaire was to be filled out through three emotions of guilt: survivor’s guilt, separation guilt, and guilt through responsibility or duty. During MRI, the participants rated levels of guilt or shame with 30 neutral, 30 guilt-, and 30 shame-related sentences. In participants with OCD, there was an increase in activation in the right precentral gyrus, right superior temporal gyrus and the right sub-gyral region. As for the healthy participants, there was activation in the left anterior cingulate, which is critical for cognitive and affective self-regulation [50]. 

Psychopathy is associated with a cluster of traits including manipulativeness, dishonesty, narcissism, superficial charm, reckless risk-taking, impulsive antisocial behavior, and arguably one of the most characteristic traits, lack of guilt [51]. Psychopaths show significant impairment in the orbital frontal cortex (OFC) and the prefrontal cortex, which are most commonly recognized as the most crucial brain structures to be compromised in violent and antisocial populations [52,53]. Yang et al. examined regional variations in cortical thickness in 27 psychopaths and 32 non-psychopaths. A structural MRI was used to determine the measurements of cortical thickness. Researchers found that psychopaths showed significant cortical gray matter thinning in the right frontal and temporal cortices in comparison to non-psychopaths. This was observed prominently in the right middle frontal gyrus, right rectal gyrus, posterior cingulate gyrus, and medial temporal cortex (including para-hippocampal gyrus and temporal pole) [53]. These findings support that disturbances in both regions play a role in emotional deficits characterizing psychopaths and the important role in the underlying neuropathology of psychopathy. 

In another study, subjects were recruited from five temporary employment agencies in Los Angeles due to this group having high rates of violence perpetration. The participants consisted of males diagnosed with antisocial personality disorder (APD), males who did not have either APD or drug/alcohol dependence, and males who had a lifetime diagnosis of drug/alcohol dependence but not APD. Using MRI, males with APD showed significant reductions in the orbitofrontal, right middle frontal gray volumes. Participants with substance abuse issues also showed significantly reduced gyral volumes compared to the control group. Reduced volumes of the right middle frontal regions help explain the lack of self-awareness of their actions and the impulsive-aggressive behavior that is found in individuals with APD [54].

In addition, mounting evidence has shown structural and functional abnormalities in antisocial individuals, and numerous findings have linked antisocial behavior to deficits in the prefrontal cortex, temporal cortex, insula, amygdala, hippocampus/parahippocampal, and anterior/posterior cingulate gyrus [55]. Seara-Cardoso et al. used fMRI with guilt-eliciting moral scenarios on male participants. The study was conducted to test if variance in psychopathic traits was associated with neural processing of anticipated guilt for personal moral transgressions. During the study, participants were instructed to imagine themselves in a specific scenario and rate how guilty they would feel. Results found that participants showed significant activation in the right amygdala, right anterior insula, and right supramarginal gyrus [56]. There was also a positive parametric modulation of feelings of guilt elicited by moral transgressions in the anterior insula. 

Responses to ameliorate feelings of guilt may reflect stereotypical response patterns and gender differences [57]. Women are reported to be more prone to guilt and shame, while men are reported to feel more trait guilt. Though guilt has been shown to function in the same way for men and women, any observed effects for women are likely to generalize to men [57,58]. In a study conducted by Amodio et al., female psychology students volunteered as participants for course credit. Using EEG, each subject viewed a series of faces that varied in race, such as White, Black, and Asian male faces with neutral expressions. A set of 12 neutral, 12 positive, and 12 negative non-face pictures were presented to the subjects as they had to remain still and focused on the photos. After the EEG, two bogus bar graphs appeared depicting the subject responding positive to the white faces and negative to the black faces. After receiving the feedback, subjects reported increased guilt, anxiety, sadness, and felt other negative effects. The subjects showed a reduction in the left side frontal asymmetry from guilt, but it was not correlated with anxiety, sadness, other-directed negative affect, or positive affect [58]. These findings supported that feelings of guilt arise from an individual’s personal transgressions, which are associated with the reduction in frontal cortical asymmetry. 

In a study conducted by Wagner et al., fMRI was used to locate the specific brain areas activated when experiencing guilt in female participants. Before the use of fMRI, each participant detailed personal past events where they experienced high levels of guilt and labeled these events with keywords [59]. This same process was also completed with the control emotions (sadness and shame). During fMRI, each participant was shown the keywords they provided for each personal event and instructed to relive the specific emotion they experienced during that time. The results revealed that there was high activation in the right lateral orbitofrontal cortex (OFC) in response to reliving guilt but not shame and sadness [59]. These results demonstrated an involvement of the OFC in guilt-related emotions; the more guilt you feel in everyday life, the greater the activation in the right OFC in situations conceived to elicit guilt [59].

Another case involves a 23-year-old Caucasian female with no history of neurological diseases or other medical issues. Before using quantitative electroencephalography (qEEG), the female participant read stories concerning an event in which she reported having guilty feelings towards and other events/crimes about which she had no knowledge. The stories were alternated by reading as false crime, true crime, and false crime to provide analysis of the changes across repetitions [60]. When the participant listened to the fake crimes, the left temporal region was found to be activated from anxiety. When the participant listened to the real crimes, guilt was shown particularly in the right temporal region [60]. These findings suggest that the participant’s response to real crimes is more associated with activity in the right hemisphere rather than the left hemisphere due to having more feelings of guilt. 

## 4. Jealousy 

When the right hemisphere sustains trauma, abnormalities often follow including: impulsivity, hysteria, depression, abnormal sexual behavior, and delusion [61]. Less frequently observed, Delusional Jealousy, also known as Othello Syndrome, has been known to occur in spontaneously arising psychoses as well as in diseases such as Alzheimer’s or Parkinson’s [62]. However, there have been increasing reports in the literature of individuals experiencing delusional jealousy following right hemispheric stroke [63]. Damage to areas in the right hemisphere as well as the frontal lobes has a major impact in these developing delusions [64]. In order for these individuals to be diagnosed with Delusional Jealousy, they must experience delusions regarding their partner’s current or past infidelity without having any evidence of such actions occurring [63]. These symptoms can arise immediately after damage or years later [65].

Psychotic symptoms, including delusional jealousy, following a stroke can range from a 1% to 5.3%; a majority of these cases occur after a right hemispheric lesion [66]. For example, a 65-year-old man admitted to the hospital underwent an MRI that uncovered a temporoparietal insular ischemic lesion. Within a week post-trauma, he began to accuse his wife of being intertwined in a sexual relationship with one of their sons, going as far as to request a paternity test of their youngest daughter due to his certainty that she was the result of an incestuous relationship [67]. His delusions made him believe that his wife was eventually going to murder him, prompting swift medical intervention in the form of fluvoxamine and chlorpromazine [67]. Symptomatic relief in the case of this patient was observed following a couple of weeks post treatment; a month follow up still found non-disruptive residual delusional activity, suggesting functional rather than anatomical abnormalities.

In 1991, a 49-year-old man became diagnosed with a right hemisphere infarction in the middle cerebral artery. He was said to have mild weakness of his left extremities, but his daily routine was still manageable. Four years later, he began to develop symptoms of hypersexuality and delusional jealousy so severe that he accused his wife of having an incestuous relationship with another relative. His delusional jealousy became so prevalent that it required him to seek help at an outpatient clinic once he turned 63 [65]. He was prescribed 50 mg of quetiapine twice daily and 10 mg of zolpidem. On his second day of treatment, his wife immediately began to notice a significant decrease in symptoms related to his delusional jealousy. After four months, she was once again able to live with him [65].

Another case involves a 20-year-old young woman who was admitted to the hospital after experiencing a hemorrhagic cerebrovascular infarction in the right parietal and frontal regions extending into the basal ganglia including the right caudate nucleus [68]. Two years later, she began a relationship with a young man and suddenly developed delusional feelings of jealousy towards her partner. The patient accused her partner of being unfaithful with their neighbors and his co-workers, even though she had no evidence of such things. After their break-up and the patient’s suicide attempt, a clinical analysis of the patient revealed right frontal lobe damage. After being treated with paroxetine consistently, the delusional jealousy subsided and did not return. 

A related case involves a 74-year-old man who was admitted to the hospital after experiencing delusional jealousy. The patient had three right hemisphere cerebrovascular infarctions within a year [69]. Following these traumas, he had delusions of his wife being unfaithful and he attacked her with a knife [69]. The patient was treated with thioridazine and pimozide daily and the delusions then subsided. 

Takahashi et al. employed fMRI to map brain activation of jealousy in healthy individuals [70]. They discovered that men and women exhibited different brain activation patterns when it came to sexual and emotional infidelity. Males showed more activation in the amygdala concerning sexual infidelity and, in the hippocampus, hypothalamus, and insula concerning emotional infidelity [70]. In turn, females experienced more brain activation in the thalamus and cortical regions in response to both sexual and emotional infidelity [70]. Comparing these data with, for example, delusional jealousy in a 77-year-old man after a right middle cerebral artery infarction [71], reveals significant overlap. Single photon emission computed tomography (SPECT) performed on the patient revealed that there was decreased blood flow to the right frontal lobe, which could result in damage to the area. These data, combined with the case of the 20-year-old woman experiencing delusional jealousy following right frontal lobe damage, suggest that the right frontal lobe plays a role in delusional jealousy [71]. 

In 1965, a 61-year-old woman had her right orbitofrontal cortex removed in order to remove a tumor in the area [72]. Thirty-six years later, the patient began experiencing delusions of her husband’s infidelity. She was convinced her husband had been unfaithful with a 70-year-old woman and they had over 10,000 children together [72]. 

In patients with non-degenerative and degenerative dementia, Othello syndrome can develop as cognitive function deteriorates [73]. During the early stages of dementia, the accusations of infidelity usually occur at night. However, as the dementia progresses, the allegations become more frequent. After performing MRI on patients with different forms of dementia who displayed Othello syndrome, results showed deterioration of gray matter in the dorsolateral prefrontal cortex [73]. Because the frontal lobes are involved in working memory and familiarity integration, as well as self-related processing, these data are supportive of self-specific emotional information errors [73]. 

We bring up a final case to demonstrate that the cases rarely involve only jealousy, but contain other classic dementia symptoms. In 2007, a 57-year-old woman had been observed by her family members to show cognitive abnormalities. She was unable to dress herself, made errors in simple housekeeping tasks, and had mild memory loss. This was soon taken to another level followed by a car crash her drunken husband had found himself in, along with huge financial debt. It was then that she began to accuse him of infidelity, despite little evidence supporting her claim. Her delusional jealousy reached the point of hospitalization. An immediate medical examination found no irregularities; however, a CT scan later discovered that she suffered from dysfunction in the right parietal lobe in relation to early-onset Alzheimer’s disease [74]. Her early-onset Alzheimer’s was treated with donepezil, and her delusional jealousy was found to have been improved [74]

## 5. Shame and Embarrassment

Given that the right hemisphere is involved in numerous evolved emotional functions including negative affective stimuli processing [75,76,77], it is tempting to think that negative self-related cognitive activity has little to do with the self and much to do with negativity [78]. However, negative emotions that are self-related appear to ‘act’ similar to positive self-emotions (i.e., pride) in that we see almost identical regions of activation [79].

When faced with negative emotional spectrums, such as shame and embarrassment, multiple imaging techniques have been employed [80]. These techniques can be utilized for both situationally induced and naturally observed emotional events. Shame and embarrassment are often correlated with activity in the orbitofrontal cortex, dorsolateral prefrontal cortex, middle temporal gyrus, and the right temporoparietal junction [81,82,83].

Duan et al. found that shame and embarrassment resulted in increased activity in the inferior temporal, anterior temporal regions, and the parietal gyrus [84], similar to those found in guilt [79,81]. Tapping into social cognition [85], it has also been found that witnessing the self-embarrassment of others activates the right middle temporal gyrus (rMTG) and the rTPJ [86]. While not tied to specific areas, shame and embarrassment have been linked to changes in oxytocin levels [87,88]. We mention this as it provides evidence of the role that these emotions play on a social and self/other level.

“Secondhand embarrassment,” also known as vicarious embarrassment, is a unique physical and behavioral social experience of susceptibility to the observation of an event or action where embarrassment elicits a bridge between physical and mental discomfort [89,90]. This includes feelings such as a sensation of internal and external cringing, usually in response to one’s own or another’s actions that do not generally conform with social norms [86]. In an event-related potential (ERP) study, the influential potential of vicarious embarrassment was examined to determine the role it plays in emotional judgment. The results revealed that vicarious embarrassment evokes negative medial frontal negativity (MFN) and N400 signals over the frontal region when faced with awkward and embarrassing situations. There was a difference for one’s own embarrassment such that if one is embarrassed (rather than observing another’s experience), there is more right hemisphere activity [91]. This nicely ties the emotion, when experienced as a self-conscious emotion to the right hemisphere, whereas seeing another’s experience (and being embarrassed for them) becomes more left-hemisphere.

Another study monitored the neural correlates of second-hand embarrassment through the use of “spinach in the teeth tasks.” In these tasks, social mishaps were displayed to observe neural judgment and concluded the study with evidence of increased activation in the rMTG and rTPJ [86], but only when an individual took the view of the other person. That is, self was required to find a right hemisphere bias, which appears to directly link the notion that it is the ‘self’ in the emotion that activates right hemisphere networks.

## 6. Pride

We do not normally think that spinach in the teeth can provide answers to neuro-emotional questions. Likewise, wearing a ‘work’ uniform is not the first thing that comes to mind when thinking about identifying brain/emotional correlates. However, using uniforms and word associations, pride in what one’s uniform represented was found to activate classic reward and empathy networks [92]. This suggests a social component to pride in which the self is promoted in the view of others. Interestingly, the involvement of the right hemisphere also involved posterior regions (e.g., rTPJ), which the researchers saw as pride involving more ToM and less ‘self’ (and therefore less MPFC activity). In other words, pride depended on knowing what others were thinking.

Similar to all self-conscious emotions such as envy [93], there is a social and evaluative component with pride. Additionally, as is the case with self-conscious emotions, pride can be adaptive or maladaptive (i.e., authentic pride vs. hubristic pride), which is what is observed in guilt, jealousy, etc. It can change over the lifespan, and it can differ according to demographics [94,95,96]. That being said, given that self-awareness is highly influenced by social factors [97], we would predict that pride would also be sensitive to ‘thinking about what others are thinking’ [98]. The above-mentioned uniform study bears this out, as do others.

For example, decoding pride (and other self-conscious emotions) involves mainly the rTPJ [99]. Critically, this occurs above and beyond basic emotional recognition. Though both hemispheres contribute to pride [79], the right hemisphere bias occurs not only functionally, but anatomically as well. For example, those high in self-esteem and pride have a greater rTPJ volume compared to those with less [100]. Grey matter differences also exist in the precuneus [101], which has been linked to self-awareness [102,103]. In this study, gratitude rather than pride was more dominant in the rTPJ. 

Considering classical fMRI studies, the experience of pride activates the rTPJ [104]. In fact, similar to the above studies, there is typically rTPJ activation at a greater rate than frontal. We find this interesting as the disruption of the MPFC leads to reductions in self-enhancement [97,105,106,107] and we would therefore expect a more consistent activation of frontal regions in studies of pride. We speculate that self-enhancement may be more similar to hubristic pride and that future studies should examine different pride types in more detail. It is possible that other factors are at play, such as the role of benign vs. malicious intent. Differences in intent appear to be important in differentiating experience and therefore one would predict differential underlying cortical correlates [93]. 

That is not to say pride does not involve frontal regions [108]. However, we assume that as ToM requirements increase, there will be a bigger pull of rTPJ [109]. To test this, we examined excessive pride in a non-clinical sample [110]. We found that one’s own excessive pride was correlated with the ability to self-recognize. Furthermore, disruption of the right frontal lobe was associated with a reduction in sub-clinical narcissism. This indicated that in a task that did not involve ToM, the frontal lobes were engaged and influenced by one’s level of excessive pride. 

## 7. Conclusions

Imagine the world without the self-awareness most humans possess. This would be a society without guilt or empathy, without shame or pride or jealousy. These societies likely exist in the animal kingdom, for example, eusocial insects are almost certainly not self-aware in any manner similar to humans (and thus lack these emotions). These animal societies exist and function quite well, yet quite differently. Ants do not build tunnels to make other ants jealous, while humans do not bat an eye bragging about the new house they purchased. We see that in humans, much of what we do is based on having these emotions. Avoiding guilt, confronting jealousy, or feeling good about the tip we just left. 

We believe the evolution of the regions detailed here provide the richness of what we consider to be everyday living for most humans. Those with experience dealing with right frontal patients often see a dulling of these emotions, both in expression and reception; as we have detailed here, it is possible to lose self-conscious emotions or have an over-expression of them. We believe that the rTPJ in particular exists to enliven and empower social interactions. We further suggest that the Fronto-Temporal networks provide a bridge between the self and affective states. Humans are unique in the richness of language, the deepness of teaching and the degree of self-awareness. The rTPJ and right Fronto-Temporal connections are critical to one’s self-awareness and turning it into ‘something useful’ by moderating emotions that adjust behavior most often in a beneficial manner.

Both the richness and difficulty of having a self are attributed at least in part to these emotions. We have only touched a small, yet vital component, which is the degree of self involved in the cognitive-affective process. However, there lies a rich literature in the development of, the evolution of, and the pathology of self-conscious emotions. We encourage interested readers to pursue what we have found to be an extremely engaging component of higher-order emotional processing. 

## Data Availability

Not Applicable.
